# Laguerre-Gaussian mode sorter

**DOI:** 10.1038/s41467-019-09840-4

**Published:** 2019-04-26

**Authors:** Nicolas K. Fontaine, Roland Ryf, Haoshuo Chen, David T. Neilson, Kwangwoong Kim, Joel Carpenter

**Affiliations:** 1 Nokia Bell Labs, 791 Holmdel Rd., Holmdel, NJ 07722 USA; 2 School of Information Technology and Electrical Engineering, The University of Queensland, Brisbane, QLD 4072 Australia

**Keywords:** Fibre optics and optical communications, Optical physics, Photonic devices

## Abstract

Exploiting a particular wave property for a particular application necessitates components capable of discriminating in the basis of that property. While spectral or polarisation decomposition can be straightforward, spatial decomposition is inherently more difficult and few options exist regardless of wave type. Fourier decomposition by a lens is a rare simple example of a spatial decomposition of great practical importance and practical simplicity; a two-dimensional decomposition of a beam into its linear momentum components. Yet this is often not the most appropriate spatial basis. Previously, no device existed capable of a two-dimensional decomposition into orbital angular momentum components, or indeed any discrete basis, despite it being a fundamental property in many wave phenomena. We demonstrate an optical device capable of decomposing a beam into a Cartesian grid of identical Gaussian spots each containing a single Laguerre-Gaussian component, using just a spatial light modulator and mirror.

## Abstract (web summary)

Previously, separating Laguerre-Gauss modes of light contained in a single beam required complex setups and could only separate a few modes. Here, the authors demonstrate a simple device capable of spatially decomposing a beam into hundreds of Laguerre-Gaussian components.

## Introduction

Arguably the next most widely used spatial basis after Fourier are Hermite–Gaussian (HG) and Laguerre–Gaussian (LG). Both are eigenfunctions of the Fourier transform, solutions of the paraxial wave equation, and eigenmodes of parabolic refractive index waveguides and quantum harmonic oscillators. LG modes are also circularly symmetric and directly related to the quantised orbital angular momentum (OAM) of photons^1–3^ and electrons^4^. For these reasons, the LG modes play an important role in many disparate areas of physics. Applications can relate directly to OAM transfer, for example, light–matter interaction inducing mechanical torque^5^, atomic transitions^6^, rotational Doppler shift^7^ or OAM imparted by astronomical objects such as black holes^8^. In other applications, LG modes are used less for their relationship to OAM, and more for their self-similar propagation properties and/or as an infinite state space for packing as much information as possible into a finite aperture or single particle, for example, quantum optics^9–11^, telecommunications^12–15^, quantum memories^16^ or incoherent beam combining^17^. Other applications such as imaging^2^ use the LG basis for spatial filtering, from microscopy^18,19^ to astronomy^20^.

Historically, the technology associated with the generation and detection of LG modes has a tendency to grow out of the field of optics^1,2,21,22^, before being extended into lower frequencies from radio to terahertz^23,24^, high frequencies such as x-rays^25^, and other wave phenomena entirely such as acoustics^26^, electrons^4,27^ and neutrons^28^. The first demonstrations, regardless of the wave type, are the ability to generate/detect LG modes one-at-a-time using a spatial filtering approach, such as a spiral phase plates or fork holograms^4,21,23,25,26,28^. In the wavelength domain, these are analogous to bandpass filters, which allow transmission of only a single band of wavelengths. Similarly in the polarisation domain, these are analogous to polarisers, allowing transmission of only a single polarisation component. As these types of filters discard all components of the wave not in a particular state, they are inherently lossy and inappropriate for many applications. Alternatively, lossless components such as dispersive gratings or polarising beam splitters spatially separate or combine the components of the beam. For example, imagine if in the spectral domain, no such lossless combination and decomposition components existed. In this scenario, components can only be split/combined with large loss, or measured one-by-one. Many applications from spectroscopy to wavelength division multiplexing would be greatly hindered or entirely unfeasible. Yet that has effectively been the scenario in much of the spatial domain.

Lossless spatial decomposition is possible in the Fourier basis using lenses, however no device previously existed capable of a full two-dimensional low loss decomposition in the LG basis for any large number of spatial components. This is despite its fundamental importance to so many disciplines, its direct relationship with OAM and approximately 25 years of research on the generation and detection of such beams. As a 2D orthogonal set, each mode in the LG basis is denoted by two indices, the radial index^29–31^, *ρ*, and the azimuthal index^1,3^, *l*, representing the topological charge and the OAM per photon. To describe an arbitrary 2D beam, both indices are required. Various mode (de)multiplexers, also called ‘mode sorters’, have been developed over the years which have implemented some limited ability to decompose a beam into its orthogonal LG components. Most approaches are able to decompose in only one-dimension, typically the azimuthal^32–34^ component, or in the past year, the ‘forgotten’ radial component^35,36^, but not simultaneous sorting of both, or in a non-orthogonal fashion^37^. These one-dimensional devices can be thought of as analogous to cylindrical lenses for Fourier decomposition in that they perform the decomposition along only a single-axis of a 2D space.

Approaches based on sorting *N* modes through a cascade of *N*−1 interferometers^32,35,36^ are inherently difficult to scale to large mode counts. A significant advance was the log-polar-based azimuthal mode-sorters^24,27,33,34,37^. Importantly, these require only a constant number of two planes of phase manipulation regardless of the number of spatial components being sorted. The device in its simplest form has some non-ideal theoretical and practical properties such as large required phase contrast per plane, and non-Gaussian mode dependent output spots. However the simplicity of the device, and the lack of alternatives, has seen it become widely used in optics, as well as other bands of the electromagnetic spectrum^24,38^ and recently for electron beams^27^.

**Fig. 1:**
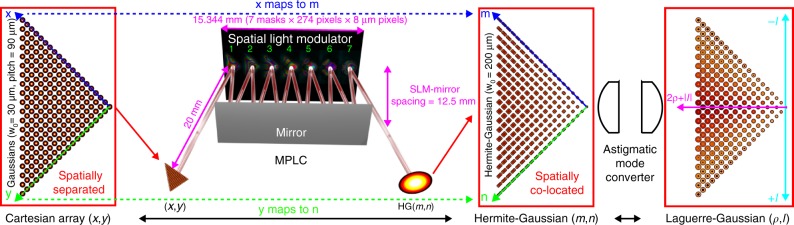
Laguerre–Gaussian mode sorter based on multi-plane light conversion. Cartesian grid of Gaussian spots (MFD = 60 μm) at positions (*x*,*y*) pass through the MPLC system, consisting of 7 phase plates separated by ~25 mm of free-space propagation, implemented using a spatial light modulator and a mirror. Through these 7 planes each input spot at position *x*,*y* is mapped to a corresponding Hermite–Gaussian mode (*m*,*n*) (MFD = 400 μm), which is in turn transformed into the Laguerre–Gaussian basis through use of two cylindrical lenses

**Fig. 2:**
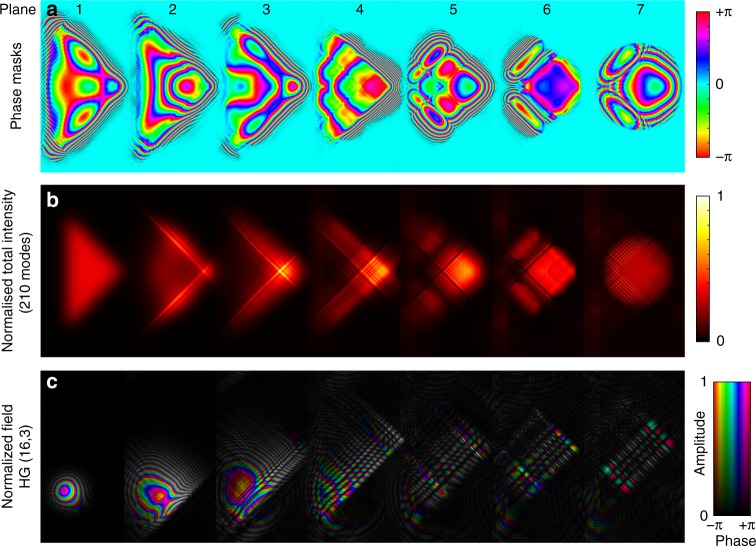
Cartesian to Hermite–Gaussian transformation using multi-plane light conversion. **a** 7 phase planes used to perform the transformation. **b** Total intensity of the first 210 modes in each plane. **c** Example of the evolution of the complex amplitude of the HG16,3 mode through the device. The physical width of these 7 masks is 15.344 mm (7 planes × 8 μm pixel pitch × 274 pixels). Although the same design can be scaled to other dimensions as discussed in Supplementary Note 8

In this work, as illustrated in Fig. 1, we have discovered that an important special class of transformation, Cartesian points (*x*,*y*) to the Cartesian indices (*m*,*n*) of HG modes, can be performed using remarkably few planes of equally space phase manipulation. The HG basis can in turn easily be transformed to LG through two cylindrical lenses^1^. Over 210 modes are demonstrated using a multi-plane light conversion (MPLC) device^39,40^ consisting of just 7 planes of phase manipulation (Fig. 2a) separated by free-space. Supplementary Figures 12–15 also demonstrate another example of 325 modes. Previously an MPLC device supporting 210 modes would have been expected to require a completely impractical 300–400 planes. Not only would so many planes be difficult to physically implement, but the cascading of even small losses per plane would easily render the approach more lossy than simply beam combining with beamsplitters or multiplexed correlation filters^22^. This device is capable of performing a two-dimensional decomposition in the HG and LG bases, as well as being the highest dimensionality mode sorter of any kind. It can be thought of as the discrete spatial analogue of a spectrometer, or the LG (OAM to real-space) analogue of a Fourier (linear momentum to real-space) lens. As was the case for the log-polar azimuthal-only mode sorter^24,27,33,34,37^, the same design can be implemented in reflection or transmission, as well as diffractively or refractively across much of the electromagnetic spectrum and more recently in electron beams, using existing technology.

## Results

### Phase mask inverse design algorithm

The masks are calculated using an inverse design process^40^, known as wavefront matching^41^, which is similar to adjoint optimisation^42^, or backpropagation in artificial neural networks^43^. The algorithm is surprisingly simple and effective, and the optimisation process has been visualised as a video available online at the address of Supplementary Note 1, and is also archived^44^. The algorithm is also provided as commented Matlab code^44^. In short, the algorithm^40^ attempts to match the phases of each pair of input and output modes at all points in space using the discrete phase planes. The masks for each of the seven planes are calculated numerically by propagating the desired basis (Cartesian grid of Gaussian spots) at one end through the optical system, and the corresponding desired output basis (HG modes) in the backwards direction. The phase masks are then updated iteratively until convergence by numerically propagating from plane-to-plane, backwards and forwards through the device. At each step, the phase mask is updated to become the phase of the superposition of the overlaps between each pair of input (*A*) and output modes (*B*). That is, the phase masks at each step become the average phase error between the modes propagating forward (*A*) and the modes propagating backwards (*B*). Specifically, the phase (*ϕ*) at each plane at each step is given by, $\phi = {\mathrm{arg}}\left\{ {\mathop {\sum}\nolimits_{i = 1}^N {A_iB_i^ \ast } } \right\}$, where *N* is the total number of modes (210 in this case), and *A*_*i*_ and *B*_*i*_ are the *i*th modes in the forward and backward direction, respectively. In a similar fashion to a Gerchberg–Saxton^45^ type approach, the algorithm implements a steepest-descent search, but it is not guided by any error function. Rather it reaches convergence by continually enforcing phase matching at each iteration step. The transformation is an approximation and is not strictly unique, although all low-loss solutions have similar features. The transformation is based largely on cubic phase manipulations, which generate Airy-like beams that are superimposed together to approximate HGs. Some illustrative examples are detailed in Supplementary Note 5.

### Experimental results for 210 mode device

**Fig. 3:**
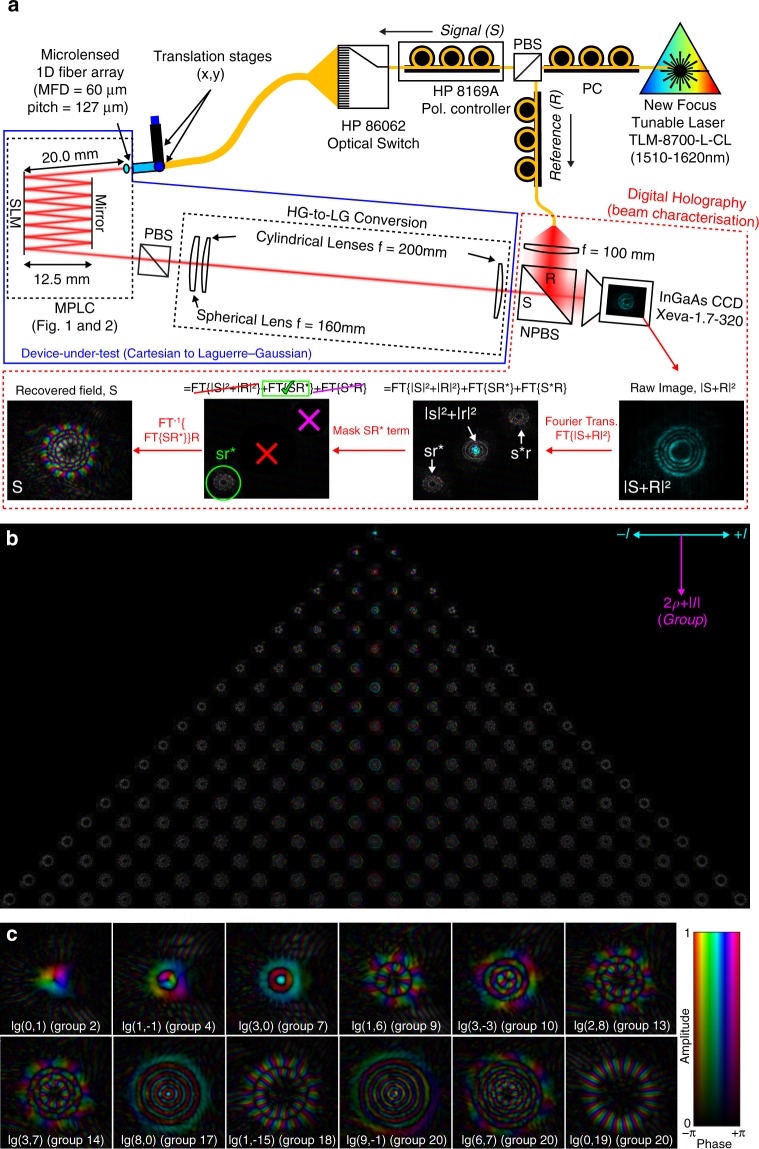
Measured optical fields at 1565 nm. **a** Measurement apparatus based on off-axis digital holography. **b** Composite image of the full set of 210 modes. Azimuthal index runs left-to-right, mode-group runs top-to-bottom. **c** Select modes of various order

The schematic of the MPLC device itself is shown in Figs. 1 and 2, with the entire characterisation apparatus shown in Fig. 3a. The device implemented here consists of an input array of Gaussian beams with mode-field diameter (MFD) of 60 μm, and a square array pitch of 127/$\sqrt 2$ = 89.8 μm. These spots propagate 20 mm before the first reflection off the SLM, a Holoeye PLUTO-II with a dielectric backplane for high reflectivity (>95%). Light is then reflected back and forth between the SLM and a silver mirror parallel to the SLM 12.5 mm away (~25 mm propagation between planes), undergoing seven reflections off the SLM, before exiting the device as HG modes with MFD of 400 μm. From there, a Fourier lens, *f* = 160 mm is used to focus the beam onto an InGaAs camera for characterisation in the HG basis, or through an additional pair of *f* = 200 mm cylindrical lenses to transform into the LG basis. Off-axis digital holography^46^ is performed to reconstruct the amplitude and phase of the output beam for each input spot in the array, for all modes over a wavelength range of 1510–1620 nm. As illustrated on the right of Fig. 2a, digital holography simply measures the intensity of the interference between the mode being measured, *S*, and a tilted reference quasi-plane wave, *R*. This intensity, |*S* + *R*|^2^, is then numerically Fourier transformed, the desired term selected, inverse Fourier transformed back into the plane of the original image, and the original tilt of the reference wave *R* removed; yielding the recovered optical field, *S*. Although performed digitally, this is analogous to physically focusing the intensity pattern |*S* + *R*|^2^ with a positive lens and picking off the desired part of the Fourier-transformed field containing information about *S*, with a pinhole aperture. The recovered field, *S*, can then be numerically overlapped with all Laguerre–Gaussian modes yielding the complex amplitude of each mode contained in that field. The advantage of digital holography in this context is not only that it captures full amplitude and phase information regarding all modes, but that it does so by adding only minimal additional optics, minimising the effect of the measurement apparatus itself on the finally measured result. Once the field is recovered digitally, all optical alignment and mode generation on the LG/HG side of the MPLC device can be essentially perfect, as this is done numerically in post-processing from theoretical ideals. Rather than having to physically implement a separate device for these operations which ultimately becomes part of the device-under-test being characterised^33–37^.

With the full output optical field recovered for every input mode, the complete linear behaviour of the device is acquired. The MPLC device is now described by an *N* × *N* complex matrix which contains the amplitude and phase of the coupling between all pairs of input/outputs modes, as a function of wavelength^47–50^. From these matrices any linear property of the device can be extracted. The matrices for both the simulated and measured device are publicly available^44^, from which the reader can calculate any linear parameter of interest. Additional detail on the experimental apparatus and procedure is available in Supplementary Note 3 and in online video^51^.

A composite image of the full set of measured optical fields at the centre wavelength for all 210 modes (20 mode groups) is illustrated in Fig. 3a. Figure 3b provides examples of various higher-order and lower-order modes of various radial and azimuthal indices and degenerate mode-group order. All modes have the correct number of rings (*ρ*) and helical phase (*l*) profiles. The full 210 mode set in full resolution is provided online, as are a HG example and a 325 mode example^44^. The results are quantified in two different bases; using the singular value decomposition (SVD) of the transfer matrix, as well as in the device’s native Laguerre–Gaussian basis. The SVD takes the transfer matrix of the device ***T*** and expresses it as the product of three matrices, ***T*** = *U*ΣV*. Where ***U*** and ***V*** are unitary transformations of the input basis (Gaussian spots) and output basis (HG/LG modes) such that **Σ** is a real diagonal matrix containing the singular values. That is, the SVD finds the input and output basis through the device such that there is no crosstalk between input and output channels, only loss. The metrics the SVD yields are independent of the basis the device was originally characterised in. The singular values are especially relevant to coherent communications employing multiple-input, multiple-output processing (MIMO) as they are related to the channel capacity. The highest and lowest singular values represent the lowest and highest loss mode superposition through the device respectively. The ratio between these two extreme singular values is the condition number of the transfer matrix, and its square is the mode-dependent loss (MDL), a measure of how ‘invertible’ the matrix is. An in-depth discussion of insertion loss (IL), MDL and the SVD is provided in Supplementary Note 2 and as an online video^51^.

**Fig. 4:**
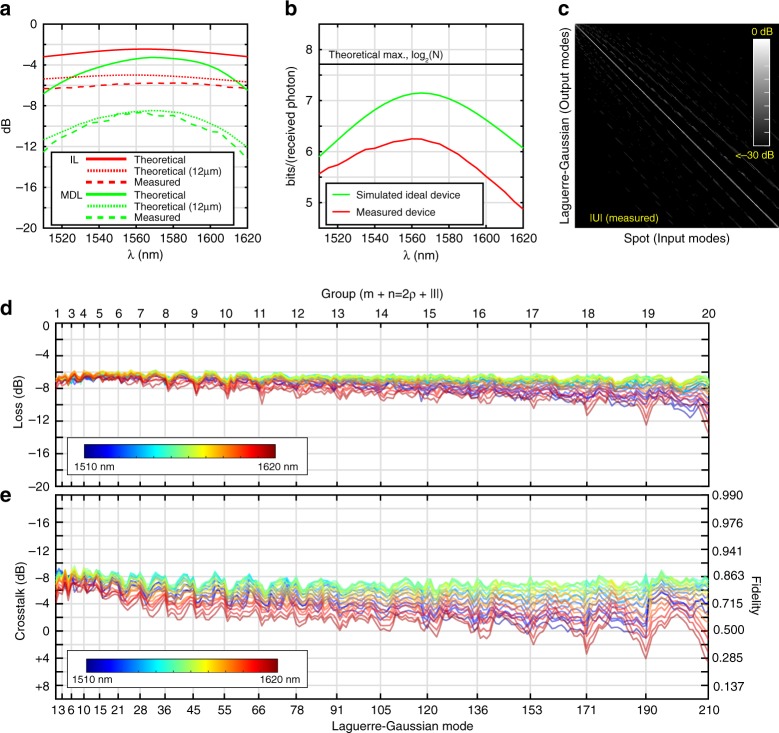
Measured properties of LG mode sorter for 210 azimuthal and radial components (20 groups). **a** Simulated and measured insertion loss (IL) and mode-dependent loss (MDL). **b** Simulated and measured information capacity per received photon. **c** Measured amplitude of transfer matrix at centre wavelength (1565 nm). **d** Measured loss of LG modes. **e** Measured total crosstalk of LG modes

The theoretical performance of the transformation is shown in Fig. 4a. IL is defined as the average loss over all possible modal superpositions through the device (average squared singular value), and MDL is the largest possible difference in loss between any two modal superpositions through the device (ratio between the largest and smallest singular value squared). Theoretically, for a lossless SLM and mirror, the transformation has an insertion loss of 2.5 dB at the centre wavelength, increasing to 3.2 dB at 1510 and 1620 nm. As all components are lossless, loss is only incurred when light is scattered into higher-order modes not supported by the system. MDL is theoretically 3.3 dB at the centre wavelength, 6.8 dB at 1510 nm and 6.5 dB at 1620 nm. Experimentally, the observed IL is between 5.8 and 6.3 dB, which for the centre wavelength corresponds with approximately 0.82 dB of total loss, or 0.49 dB of excess loss per reflection from the SLM. MDL was measured to be between 8.7 dB at the centre wavelength, 12.5 dB at 1510 nm and 13.3 dB at 1620 nm. Again, it should be noted that MDL is not the difference in loss between the maximum and minimum loss LG mode of the device. Those losses, the diagonal elements of the transfer matrix (Fig. 4c) are illustrated in Fig. 4d. The maximum variation in loss between any two LG modes is 1.7 dB at the centre wavelength. Loss and crosstalk at the centre wavelength does not strongly depend on the order of the mode group, but the overall loss and crosstalk levels do depend on the total number of modes supported. Wavelength dependence does tend to get worse for higher-order modes as these modes contain higher spatial frequencies and must diffract over a larger path length from the edges of the Cartesian array. Similar principles apply when transformations are calculated to support increasing number of modes. As mode count increases, the performance of all modes tends to degrade together as a whole, but there is more degradation in bandwidth than there is in overall performance at the centre wavelength. Discussion and examples on this topic available in Supplementary Note 5 and as an online video^51^. Experimentally, inter-pixel crosstalk^52^ on the SLM that induces blurring particularly during phase wraps is the dominant contributing factor to MDL. Example simulation results that convolve the phase level of each SLM pixel with a Gaussian of width 12 μm are illustrated in Fig. 4a for reference.

The measured transfer matrix of Fig. 4c, yields a channel capacity, shown in Fig. 4b of 6.25 bits/photon compared to the simulated ideal device of 7.15 bits/photon, or absolute theoretical maximum of log_2_(210) = 7.71 bits/photon. The worst LG mode at the centre wavelength has a total crosstalk of −5.5 dB, defined as the power in the desired mode relative to the total power in all other modes. Average total crosstalk over all 210 modes is −7.2 dB. Crosstalk per mode is the above values divided by 210, yielding −28.7 and −30.4 dB for the worst-case and average, respectively.

We have demonstrated an MPLC-based mode-sorter supporting the first 210 modes in the LG basis using just an SLM and a mirror. This device can be easily implemented using common optical components and allows the spatial properties of light to be decomposed in 2D and with high dimensionality, enabling functionality in the spatial domain, which is already common in the spectral and polarisation domains. This device could also be translated to other wave phenomena such as electrons where it could be used as a ‘spatial spectrometer’ to analyse the quantised OAM spectrum and spatial state of a particle in two-dimensions.

## Supplementary information

Supplementary Information

## Data Availability

Simulated and measured wavelength-dependent transfer matrices are publicly available as Matlab files for both 210 and 325 mode devices. As are pre-calculated phase mask sets which can be used as-is, for several common SLM models currently on the market. These pre-calculated masks can be easily scaled to other dimensions and wavelengths without recalculation as discussed in Supplementary Note 8. Full resolution images corresponding to Fig. 3b and Supplementary Figures 9, 10 and 12 are provided^44^ (10.14264/uql.2019.81).

## Code availability

Example commented Matlab code is included to demonstrate the wavefront matching procedure. Matlab code is provided to resize pre-calculated masks for different dimensions and/or wavelengths. An Excel spreadsheet is also included to assist in translating existing pre-calculated designs to other dimensions and wavelengths^44^ (10.14264/uql.2019.81).
