# Supplementary material for: Laguerre-Gaussian mode sorter
Source: Nat Commun. 2019 Apr 26;10:1865. doi: 10.1038/s41467-019-09840-4 (PMC6486581; doi:10.1038/s41467-019-09840-4)
Supplement: Supplementary file 1 — Supplementary Information [file 41467_2019_9840_MOESM1_ESM.docx]

**Laguerre-Gaussian mode sorter**

**Supplementary Information**

Fontaine *et al.,*

**Supplementary Note 1 : Videos**

A video is included which complements the content of the document itself. A 60 minute detailed technical summary of all work presented in the main document and Supplementary Material.

Detailed Technical Summary (60 mins) : <https://youtu.be/TqAr4UFtoqw>

**Supplementary Note 2 : Background Information**

**Singular value decomposition (SVD), insertion loss and mode dependent loss**

All the devices simulated and measured in this document have been quantified in terms of the complete optical transfer matrix of the device. This is a complete linear description of the device by which any input can be mapped to any output in terms of amplitude and phase. It is the only means by which parameters such as insertion loss and mode dependent loss can be extracted and the most rigorous method of characterising such components. More common methods of characterisation based on power-only measurements cannot yield a true measure of the number of spatial channels a device supports.

*Simple example from the N=2 polarisation case (polarisation dependent loss, PDL)*

The simplest way to illustrate this concept is to consider a measurement of the MDL (which in this case is PDL) of a perfect linear polariser orientated at 0^o^. If measured naively by measuring the ratio between the transmitted power of input -45^o^ and +45^o^ polarised light respectively, the mode dependent loss would appear to be 0dB, as both -45^o^ and +45^o^ have the same 3dB loss. Hence it could be incorrectly assumed that the device has 3dB insertion loss and no mode dependent loss. However there is in fact only a single polarisation state which can survive transmission through the polariser. The true mode-dependent loss is infinite, not zero, and if attempting to transmit information through the polariser on orthogonal channels, there is only a single channel of information that is supported, not two. In fact, any value of MDL between 0dB and ∞dB could be obtained depending on the measurement basis if the measurement was performed in incorrectly in this way.

However if the Jones matrix of the polariser is measured, and the singular value decomposition (SVD) performed on that Jones matrix, it would be found that the device has two transmission axes, one with 0dB loss and the other with infinite loss, hence yielding the true value of MDL. Importantly, the values of IL and MDL extracted from a SVD are independent of the basis the measurement was performed in. The singular value decomposition transforms the input and output bases respectively such that there is no crosstalk between the input and output, only excess loss (the singular values). The ratio between the maximum and minimum singular values represents the maximum possible difference in loss between any two spatial states through the device, representing the worst possible case, but is also an indication of the true number of spatial modes the device supports. For the 210 modes discussed in the main document, the maximum difference in transmitted power for any two LG modes was less than 2dB at the centre wavelength, whereas the true measured mode dependent loss is in fact 8.7dB as the highest and lowest loss modes through the system are not LG modes.

Ultimately the final measured value of MDL is a combination of the MDL of the device itself, but also the measurement apparatus used. Obtaining low values of MDL experimentally is a difficult task in its own right, as any measurement imperfection will ultimately show up in the SVD, which always finds the worst-case. Even when measuring devices which are known to have almost no MDL, such as short lengths of multimode fibre, values less than 10dB can be difficult to obtain. For instance, the full matrices for the 72-mode fibre previously discussed^1^ have measured MDL over 12dB, despite the fact that the true MDL would be closer to zero. For the 210 mode device presented here, an MDL measurement of 8.7dB is an excellent result, considering similar values are common even for devices which support an order of magnitude less spatial channels.

**Supplementary Note 3 : Characterisation procedure and apparatus**

**Supplementary Figure 1 | MPLC characterisation apparatus.**

*Characterisation apparatus description*

Shown in Figure S1 is the experimental apparatus used to characterise the wavelength-dependent optical transfer matrix of the MPLC system. The characterisation procedure consisting of feeding light in from the Cartesian spot array side, one spot at a time, and measuring the optical field for each corresponding LG mode using digital holography on a CCD camera. The characterisation yields a complete linear description of the device as an N×N complex matrix from which any linear property can be extracted and the outcome of any linear experiment predicted^1–4^. Off-axis digital holography^5,6^ is employed for analysing the spatial amplitude and phase of the LG/HG beams which allows much of the optical alignment, mode generation and overlaps to be performed digitally in post-processing from theoretical ideals. This makes the characterisation procedure very accurate as it allows the MPLC device to be measured with minimal contribution to the performance from the measurement apparatus itself. This procedure is also visualised in the Supplementary Video.

The light source is a New Focus TLM-8700-L-CL which is tunable in the range of 1510-1620nm. Light from the source travels through a manual polarisation controller (PC) before passing through a fibre-based polarising beamsplitter (PBS). The PC here is used to change the ratio of power in what will become the ‘signal’ and ‘reference’ beams of the digital holography reconstruction of the field. Once set, this ratio does not change during the characterisation procedure itself. The port of the PBS that is to become the reference beam for the digital holography reconstruction passes through a polarisation controller (PC) before being collimated on the camera using a *f*=50mm achromatic lens to produce a large (compared to the MPLC modes being characterised) Gaussian beam which acts as the quasi-plane-wave reference beam. The other port of the PBS will become the signal beam for digital holography and will pass through the MPLC system itself. After exiting the PBS in this signal arm, light travels through a HP 8169A computer-controlled polarisation controller and a HP 86062 optical switch. Each of the 25 single-mode fibres from the input microlensed fibre array are attached to the first 25 ports of the optical switch. The switch and computer-controlled polarisation controller are used in tandem such that whenever a particular port is selected on the switch, the appropriate polarisation transformation is also applied on the polarisation controller such that light for all fibres in the array enter the MPLC system in the correct polarisation. The fibre array itself is a 1D V-groove array with fibres on a standard 127μm pitch and is mounted on a translation stage with computer-control of the *x* and *y* axes. Starting at the highest-order group, to characterise the modes within a degenerate mode-group, the corresponding port on the optical switch is selected for each of the modes in turn. The translation stage then moves 63.5μm in the *x*-direction to the next mode-group, and +/-63.5μm in the *y*-direction (axis of the 1D fibre array). Hence the maximum mechanical distanced travelled in the *x*-direction will be (*N*-1)×63.5μm, where *N* is the number of mode-groups being characterised. The maximum travel in the *y*-direction will always be 63.5μm regardless of the number of mode-groups as most of the translation along this axis is implemented by switching amongst the fibre array rather than mechanical movement.

Light then passes through the MPLC system itself. The distance from the waist of the beams exiting the fibre array to the first plane on the spatial light modulator (SLM) is approximately 20mm. Light is incident at an angle of 5.011^o^, which is the angle required to produce exactly 7 bounces off the SLM given the spacing between phase masks on the SLM (274 pixels = 2.192mm) and the SLM-to-mirror distance (12.5mm). The SLM is a Holoeye PLUTO-II with a high-reflectivity dielectric mirror backplane. The SLM has a resolution of 1920×1080 with a pixel pitch of 8μm. The mirror is a silver mirror cut to the appropriate width of 6×274 pixels=13.152mm.

After exiting the MPLC system, light passes through a polariser before entering the astigmatic mode converter used to convert the HG modes coming natively from the MPLC system, into the desired LG modes. The two *f*=200mm cylindrical lenses are (√2)×200mm apart with the waist of the *f*=160mm spherical lens which is positioned between them. Some additional defocus and astigmatism aberration correction is applied on the final mask of the MPLC system to compensate for misalignment in the astigmatic mode converter.

After the astigmatic mode converter, the light from the MPLC system is then interfered with the reference wave on the CCD camera. Digital holography is performed to reconstruct the full optical field, and then overlapped numerically with each mode in the Laguerre-Gaussian basis to extract the complex amplitudes of each modal component. For a given input, this becomes a single column of the measured transfer matrix, and when combined for all input modes, becomes the full transfer matrix, which is also measured as a function of wavelength for each mechanical step of the translation stage. With the complete transfer matrix of the device specified for every input and output mode, in terms of both amplitude and phase, we now have a complete linear description of the device from which any linear property can be extracted. The linear relationship between the input and output is completely specified and any superposition of modes at one end of the device can be directly mapped to the corresponding superposition of modes on the other side. This is the same idea as the characterisation of other linear reciprocal *N*-port devices in other fields of science, such as in electrical engineering. Once the component is completely specified numerically, linear algebra techniques such as the singular value decomposition (SVD) can be performed, to find parameters such as mode dependent loss (MDL), which would be impractical and inaccurate to attempt to find physically given the enormous search space of a device supporting hundreds of modes. For example, if each mode can have *m* values of complex amplitude, and there are *N* modes, then the search space is *m^N^*. Even for the coarsest minimum case of *m*=2, our 210 mode device would have 2^210^=1.65×10^63^ possible states. However as the device is linear, it can simply be described by an *N*×*N* matrix, that only requires *N* optical field measurements (camera images) in this experiment.

*The advantage of digital holography for characterisation*

The advantage of analysing the HG/LG beams of the device using digital holography, is that there is very little source of error. It is a very general class of problem, that whenever the performance of a device is being characterised, it is important to minimise, or calibrate out, the contribution to the performance of the characterisation apparatus itself. From Figure S1 and Figure 3a, it can be seen that almost no additional optics must be added to the system. Another advantage of digital holography and physically propagating from the Cartesian spot side to the HG/LG mode side, is the HG/LG beams are generated and overlapped numerically in post-processing using the ideal theoretical beams. Hence the final measured data has minimal contribution from the measurement apparatus itself, and the MPLC device-under-test is observed almost entirely in isolation. The digital holography system adds only a single lens (for the reference beam) and a beam splitter to the output side of the MPLC device. Once the raw image is captured on the camera, all further alignment, mode generation and overlaps are performed digitally and hence can be theoretically perfect. This makes it more accurate than attempting alternate approaches based on installing an additional SLM and accompanying optics at the output of the MPLC device^7–10^. In such a case the SLM performs correlation measurements, or back propagates the LG/HG modes through the device towards the SMF array side. Although this technique would work, it would be slower and more importantly, would be sensitive to the quality of the modes generated by the SLM, the aberrations of the additional optics, and particularly the alignment of the SLM system relative to the MPLC device-under-test. The final measurement would be a characterisation of the system as a whole. With a possibly non-negligible contribution from the measurement apparatus itself, especially if the performance of the MPLC device is high. By feeding light into the system from the SMF side, no modes need be generated, only power fed into the SMF ports. Any inaccuracies in the spot generation on this side due to microlens aberrations, or pointing errors, are genuinely part of the optical system being characterised, and hence should not be calibrated out of the performance.

**Supplementary Note 4 : Discussion on the difficulty of spatial decomposition**

So why is spatial decomposition so difficult when compared to decomposing wavelength or polarisation? Why despite some 25 years of research^10,11^ on the measurement and generation of LG beams, has no device previously been able to perform a true two-dimensional decomposition? Lossless separation of wave components, whether wavelength, polarisation or space, are always performed through phase manipulation and interference. Hence the ease at which components of a beam can be spatially separated depends upon the ease at which a different phase can be applied to each component. For wavelength and polarisation this is straightforward. Wavelength-dependent phase shifts can be applied through optical path length differences, and polarisation dependent phase shifts can be applied through birefringence and/or reflections, and this spatial phase is transformed to spatial separation through propagation. Importantly, imparting spatial phase does not change the wavelength or the polarisation. In the spatial domain, not only is there no straightforward way in general to apply independent differential phase to spatial components which occupy almost identical space, but application of spatial phase changes the spatial components of the beam as it propagates. Unlike wavelength or polarisation, the input and output spatial bases must evolve through the transition, requiring in general a three-dimensional refractive index profile.

A photonic lantern^12,13^ is one example of a device which can decompose a beam into spatially separated components. It achieves this through an adiabatically tapered refractive index profile which transitions from a multimode fibre at one end, to an array of single-mode fibres at the other. However such a device is difficult to scale losslessly to large mode counts, particularly if the mapping of input modes to output spots must be controlled. A multi-plane light conversion system^14^ (MPLC) comprises multiple phase plates separated by free-space propagation and in some ways emulates a discretised version of the continuous photonic lantern but is more amenable to implementing arbitrary spatial transformations. In the general case, the number of phase planes required scales as twice the total number of spatial modes, again making large mode counts difficult in practice. However not every kind of spatial basis transformation requires this full 2*N* phase planes, and some important modal decompositions and transformations require far less. Conversion between HG and LG requires only two cylindrical lenses^11,15–17^, and log-polar-based azimuthal mode-sorters^18–20^ also require just two planes of phase manipulation regardless of the number of modes. Of course a lens was another example, performing a Fourier decomposition using a single plane of parabolic phase.

**Supplementary Note 5 : Theoretical Examples**

*Four-plane, 45-modes, Fourier-transform between each plane.*

This example serves to illustrate some of the key aspects of the transformation, by using a small number of planes separated by exact Fourier transforms (as opposed to free-space propagation between planes). In this simplified example, planes 1 to 3 have been constrained to contain only linear (tilt), focus (quadratic) and cubic phase. This simple conversion generates Airy-like beams with approximately the correct aspect ratio and lobe count to approximate the desired Hermite-Gaussian modes in one quadrant. However with just 4 planes, the resulting modes are still mostly asymmetric and Airy-like, consisting of a single dominate lobe, rather than the 4 (or 2 or 1 depending on the type of mode) equally dominate lobes of a Hermite-Gaussian. For this simple transformation example, there is still quite a lot power in spurious lobes along the *x*-*y* axis as can be seen in Figure S2d. With the addition of more planes in later examples, these spurious lobes are merged together and then into the main beam to form the missing lobes of the HG modes.

**Supplementary Figure 2 |** Total intensity of all modes (top) and phase masks (bottom) for simple four-plane, 45-mode example, where each plane is separated by a Fourier transform. Green (top) and white (bottom) spots indicate the centre-of-mass of each mode in each plane. **a,** Each spot in the original Cartesian grid is tilted such that the spots take the position of the maxima of Hermite-Gaussians in the next plane (Plane 2). **b,** Each spot is now located at a position (*x*,*y*) given by the maxima of the Hermite-Gaussian modes and with tilts equally spaced on a grid in *k*-space (due to their equally spaced positions in real-space in the previous plane). This mask is dominated by a cubic phase term. This cubic phase converts the Gaussian spots to Airy-Gauss beams. The offset of each beam in *x* and *y* creates additional focus in *x* and *y* which in turn sets the aspect ratio of the beams in the next plane. An overall quadratic phase is also part of the mask with a strength such that all modes have almost the same centre of gravity in the next plane. **c,** Cubic phase is again applied which causes the elongated beams generated by Plane 2 to become Airy-like beams in Plane 4 which begin to approximated Hermite-Gaussian beams. The beams currently have a single dominate lobe, and a decaying series of side lobes. **d,** Residual phase difference between the Airy-like beams and the desired Hermite-Gaussian basis. Adjacent lobes in the Airy-like beams are phase-flipped where appropriate to begin to group Airy-like lobes together to form larger Hermite-Gaussian lobes.

*101-plane, 210 modes, 1.7mm free-space propagation between planes*

This example illustrates how the transformation is performed when using a very large number of very closely spaced planes. Effectively the opposite scenario to that illustrated above in Figure S2. The transformation itself is illustrated below in Figure S3. It has the same total device length as the 7-plane example of the main document, but has been divided into many more planes. In this case, the transformation is more adiabatic with only small phase perturbations applied at each plane. This scenario is more like the continuous three-dimensional refractive index profile of a device like a photonic lantern^7^, and less like the discrete phase planes of an MPLC device. With so many planes, the transition is smooth leading to high bandwidth and can be made with IL and MDLs which approach 0dB. The transition itself also shows features which are present in the 4-plane example above, and all other solutions, whereby a dominant quadrant is created approximating part of the Hermite-Gaussian beams, with the additional lobes created along the *x*-*y* axis being successively merged together to form all the necessary lobes of the Hermite-Gaussian modes.


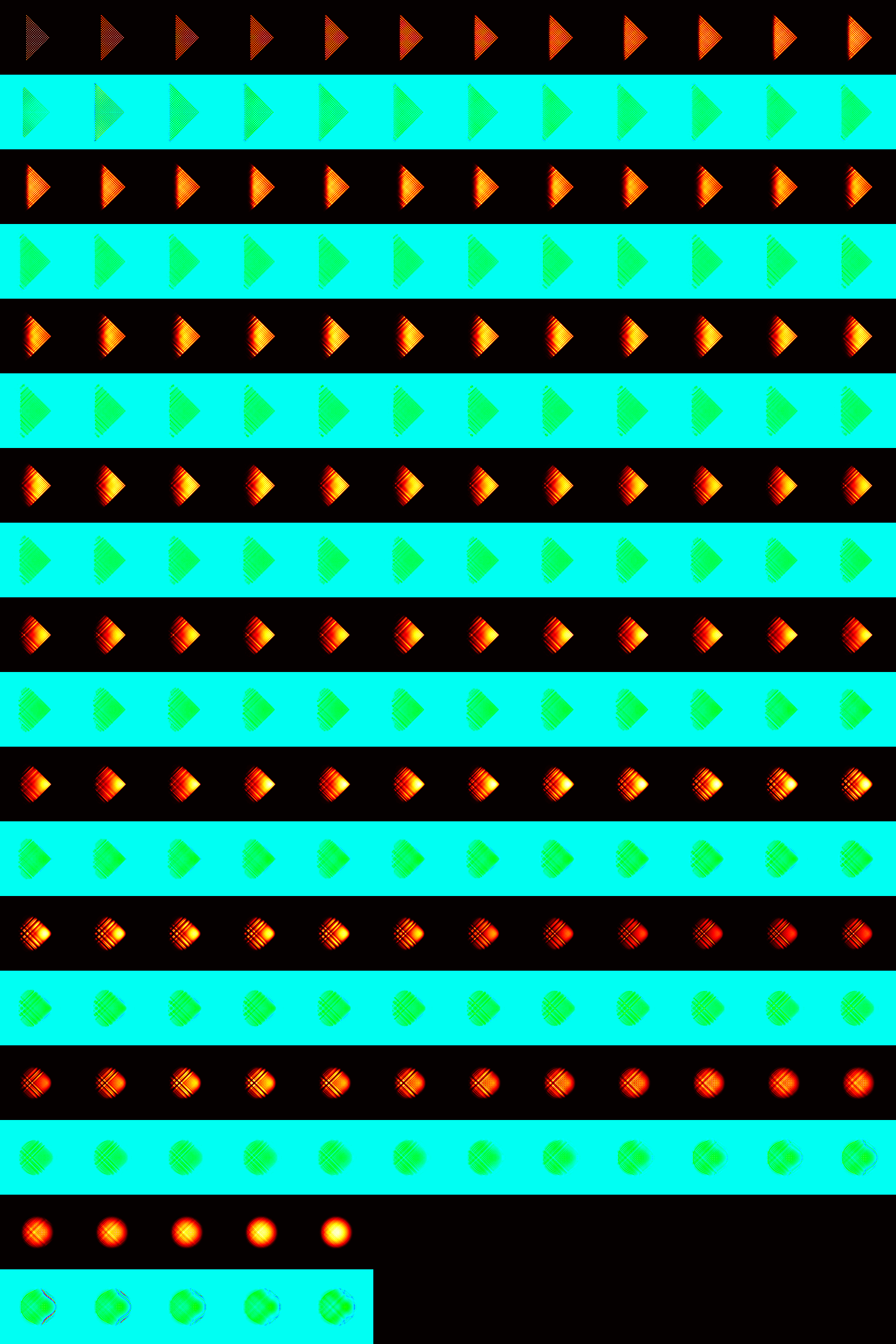


**Supplementary Figure 3 |** Adiabatic example. 210 modes, 101 planes, 1.7mm plane spacing. Intensity of all modes with corresponding phase masks for all planes. Animation of this transformation available online^21^.

*Dependence of performance on plane count (210 modes, fixed total length of device)*

In this example, devices are simulated which have the same input and output basis as the device in the main document, but with a varying number of planes. The total length from the waist of the beams coming from the input fibre array to the final phase plane is set at a fixed total path length, which is the same as the device in main document (170mm=20mm + 6×25mm). This fixed length is divided amongst a varying number of planes. Figure S4 illustrates the dependence of insertion loss and mode-dependent loss as a function of wavelength and total plane count. It can be seen, particularly from the mode-dependent loss of Figure S4b, that beyond 7 planes, there are only moderate improvements to MDL and overall bandwidth. It should be noted, that the results presented here have been optimised, somewhat arbitrarily, to minimise MDL across the bandwidth 1510-1620nm, and different plots would be obtained if other performance measures were chosen such as maximum possible IL, MDL, a different bandwidth etc. However the general trend of diminishing returns beyond approximately the 7^th^ plane is a consistent feature and the reason 7 planes were selected for the experimentally measured devices. The same trend is also true of device designs whereby the plane spacing is fixed and the total device length increases as plane count increases. Issues of practicality aside, ultimately, if a sufficiently large number of planes are included, IL and MDL can be made arbitrarily low and bandwidth arbitrarily wide. It should be noted that the results presented here are intended to illustrate the general trend with respect to total plane count, rather than an indication of the maximum possible performance for a given number of planes. For any given plane count, additional tweaking of parameters and more in-depth solution space searches could lead to better results.

**Supplementary Figure 4 |** **Simulated properties of 210 mode transition of fixed total length (170mm) and various total plane counts** **a,**  Insertion loss (IL) as a function of wavelength and plane count. **b,** Mode dependent loss (MDL) as a function of wavelength and plane count.

*Dependence of performance on total mode count*

**Supplementary Figure 5 |** **Theoretical performance of 7-plane device for various mode counts. a,** Insertion Loss (IL) and, **b,** mode-dependent loss (MDL).

Figure S5 illustrates the dependence of the insertion loss and mode dependent loss for a system with the same design parameters as that demonstrated in the main document, but for various total mode counts. A new set of phase masks is calculated optimised for each total mode count. As can be seen from Figure S5a, there is only a weak-dependence of IL on mode counts beyond the lower-order modes. However this is not particularly instructive, as IL is the average loss of all possible spatial modes through the system and not an indicator of the number of spatial modes the device supports. The mode dependent loss plot of Figure S5b is more relevant as it describes what performance can be achieved for a desired number of supported modes. It can be seen that the peak MDL at the centre wavelength degrades as the mode count increases, but the bandwidth degrades faster. The higher spatial frequencies of the higher-order modes in conjunction with the physically larger dimensions over which the light must be diffracted from the input SMF array give rise to increased wavelength dependence. Although not a constraint for the simulations in Figure S5, beyond approximately 25 mode-groups (325 modes) the SMF array dimensions using the specified MFDs (60μm) and pitch (89.8μm) used in this work begin to exceed the 274 pixel width of a single mask on the SLM.

**Supplementary Note 6 : Experimental Examples**

This section summarises the simulated and measured properties of two different MPLC designs. The first (Figures S6-S11) is the same 210-mode device presented in the main document. The second (Figures S12-S15) is for a 325-mode device.

The graphs are positioned on the page such that pressing page-up and page-down allows the reader to flip back and forth between simulated and measured results for easy comparison Figures S6-S8 and Figures S12-S13). The appropriate phase masks for these devices are available in the Supplementary Material and have been included for several common models of SLM on the market at time of publication, from Holoeye, Meadowlark and Hamamatsu. All figures follow the same format, summarised below.

*Singular value decomposition of the full transfer matrix*

**a,** Insertion loss (red) and mode dependent loss (green) as a function of wavelength.

**b,** Singular values as a function of wavelength. The mean of these values is the insertion loss, and the ratio between the first and last singular value yields the mode dependent loss

*Properties in the Laguerre-Gaussian basis for the full mode set*

**c,** Loss of each Laguerre-Gaussian mode.

**d,** Total crosstalk between Laguerre-Gaussian modes. The ratio of the power in the desired LG mode to the sum of the power in all other modes.

*Properties of device as total number of degenerate mode groups increase (using the device for less than the full number of modes it can support)*

**e,** Insertion loss as a function of total number of mode groups.

**f,** Mode dependent loss as a function of total number of mode groups.

**g,**  Total crosstalk between Laguerre-Gaussian modes as a function of total number of mode groups

**Supplementary Figure 6 |** Simulated results of 210 mode device.

**Supplementary Figure 7 |** Measured results of 210 mode device.

**Supplementary Figure 8 |** Simulated results of 210 mode device where the phase level of each pixel has been blurred to simulate pixel crosstalk on the SLM. In this case, the 8μm pixels on the SLM have been convolved with Gaussians of MFD=12μm.


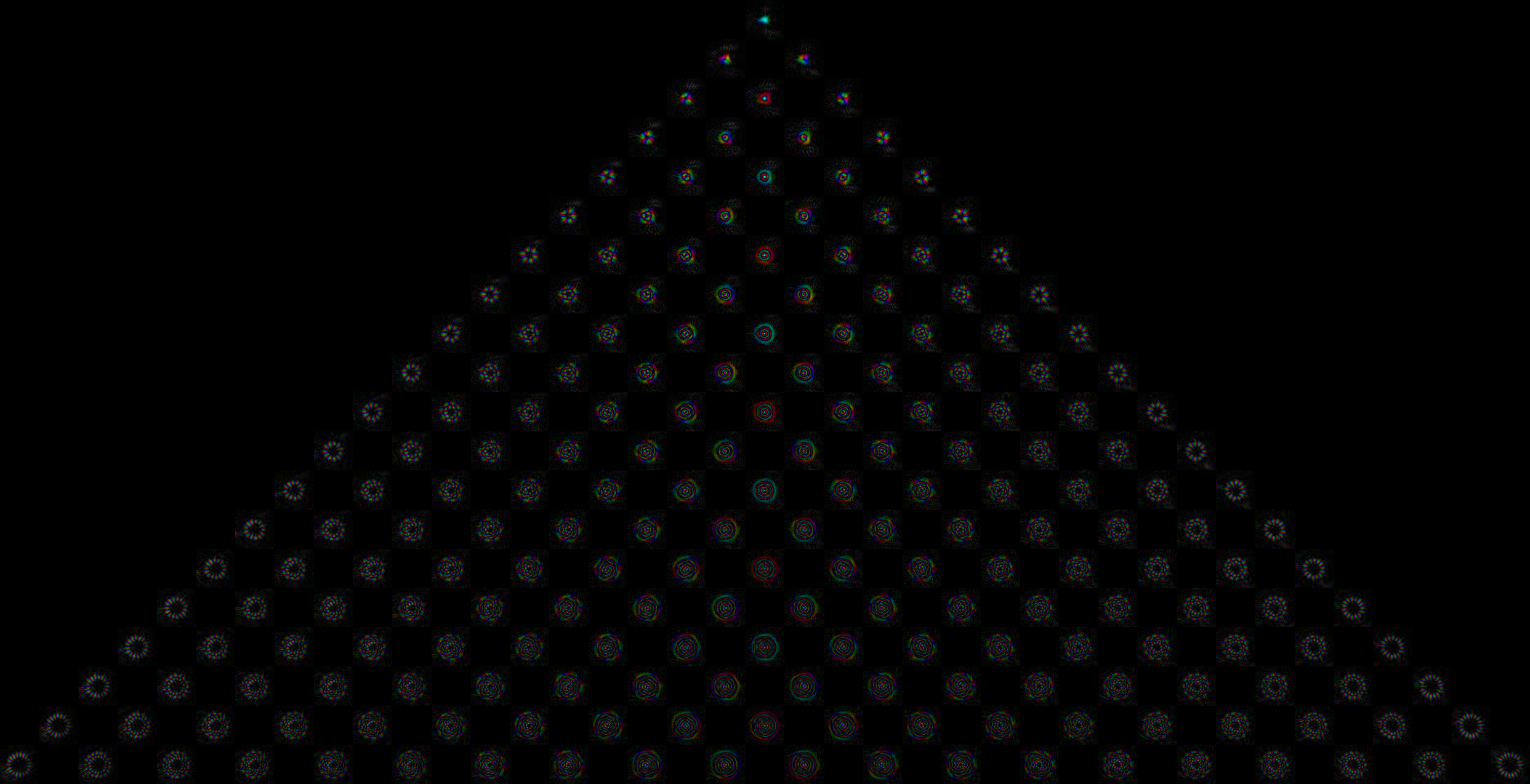


**Supplementary Figure 9 |** Measured fields for all 210 Laguerre-Gaussian modes at 1565nm. *x*-axis of the image represents ±*l*  and *y*-axis represents *ρ*. Full resolution image available in the Supplementary Material.

**
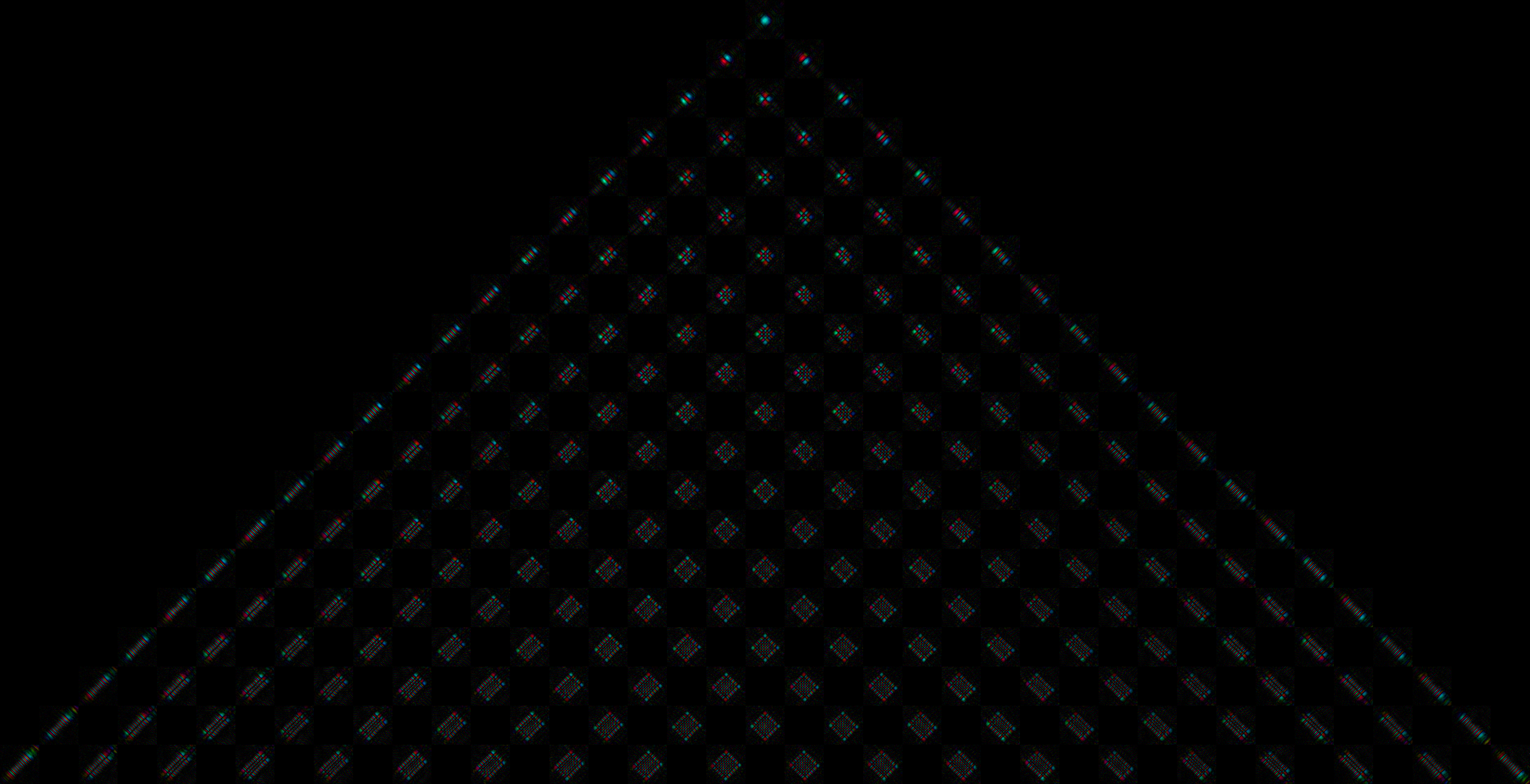
**

**Supplementary Figure 10 |** Measured fields for all 210 Hermite-Gaussian modes at 1565nm (astigmatic mode converter removed from setup). Full resolution image available in the Supplementary Material.

**Supplementary Figure 11 | Bits/photon and coupling matrices for 210-mode device. a,** Bits per received photon as a function of wavelength for ideal simulated device, and experimentally measured device. **b,** Bits per received photon as a function of total mode count used for the same device. **c,** Ideal simulated device coupling matrices at the edge and centre wavelengths **d,** Measured coupling matrices at the edge and centre wavelengths.

**Supplementary Figure 12 |** Simulated results of 325 mode device.

**Supplementary Figure 13 |** Measured results of 325 mode device.


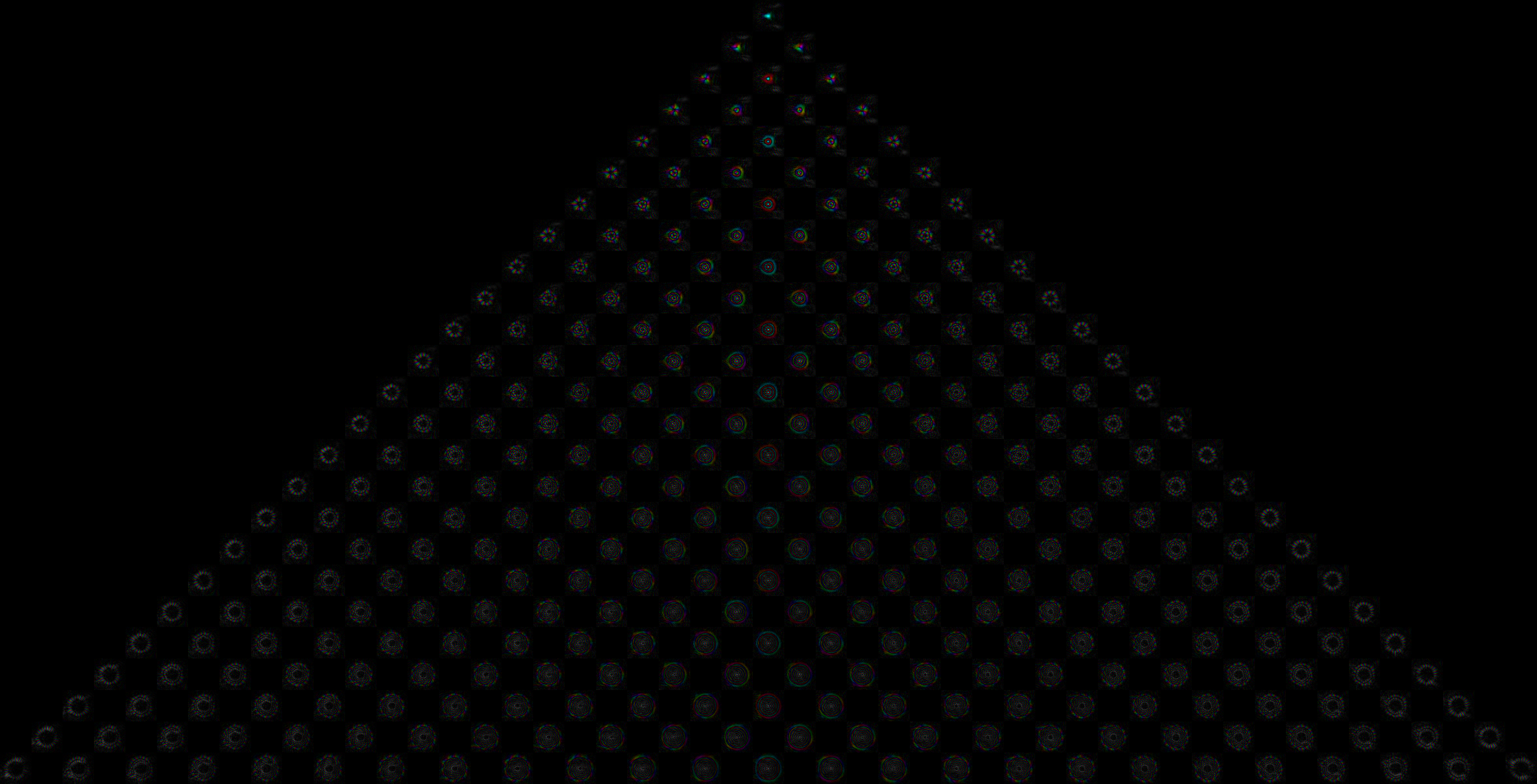


**Supplementary Figure 14 |** Measured fields for all 325 Laguerre-Gaussian modes at 1565nm. *x*-axis of the image represents ±*l*  and *y*-axis represents *ρ*. Full resolution image available in the Supplementary Material.

**Supplementary Figure 15 | Bits/photon and coupling matrices for 325-mode device. a,** Bits per received photon as a function of wavelength for ideal simulated device, and experimentally measured device. **b,** Bits per received photon as a function of total mode count used for the same device. **c,** Ideal simulated device coupling matrices at the edge and centre wavelengths **d,** Measured coupling matrices at the edge and centre wavelengths.

**Supplementary Note 7 : Pre-calculated masks for common SLMs**

Provided in the Supplemental Material are pre-calculated masks for the 210 mode device used in the main document. These masks are available as a Matlab file (HG210_PLUTOII.mat) which contains the masks themselves, as well as pre-calculated greyscale PNG image files containing the entire mask set, correctly spaced, which can be immediately displayed on an SLM as-is to implement the device. The system parameters for each are shown below in Table 1. Each design has been configured such that the mask spacing is equal to the pixel width of the SLM/7.

| ***Model*** | ***Dimensions (pixels)*** | ***Pixel Size (μm)*** | ***Angle of incidence*** | ***Mask Spacing***  ***(pixels)*** | ***SLM-to-mirror spacing (mm)*** | ***Optimal Mirror width (mm)***  ***(pixel size × mask spacing × 6)*** | ***Filename*** |
| --- | --- | --- | --- | --- | --- | --- | --- |
| **Holoeye PLUTO II** | **1920×1080** | **8** | **5.011^o^** | **274** | **12.5** | **13.1520** | **HG210_PLUTOII_1920x1080_8um.png** |
| Holoeye GAEA | 3840×2160 | 3.74 | 4.687 | 548 | 12.5 | 12.2971 | HG210_GAEA_3840x2160_3.74um.png |
| Meadowlark 1920×1152 | 1920×1200 | 9.2 | 5.758 | 274 | 12.5 | 15.1248 | HG210_Meadowlark_1920x1200_9.2um.png |
| Hamamatsu X10468 | 800×600 | 20 | 5.211 | 114 | 12.5 | 13.68 | HG210_Hamamatsu_X10468_800x600_20um.png |
| Hamamatsu X13138 | 1280×1024 | 12.5 | 5.20 | 182 | 12.5 | 13.65 | HG210_Hamamatsu_X13138_1280x1024_12.5um.png |

**Supplementary Table 1 |** Summary of Supplemental pre-calculated SLM masks for different SLM models.

Furthermore, a Matlab script is included (maskInterpolate.m) which can be used to interpolate the pre-calculated masks for use on a different SLM, or for a different mask spacing. Say for example, spacing the masks slightly closer for use with a standard 12.5mm or 12.7mm mirror.

**Supplementary Note 8 : Using pre-calculated masks at different wavelengths and/or dimensions**

The work presented here has been performed at a centre wavelength of 1565nm. However it is straightforward to use these masks at other wavelengths simply by scaling the propagation distance between planes. A shorter wavelength means a longer distance is required between planes such that the equivalent diffraction occurs. Halving the wavelength, doubles the distance required between planes. Below in Table 2 are some examples of the parameter changes required as the wavelength is altered. For the device experimentally demonstrated in this work, the plane-to-plane spacing is 25.096mm, which consists of twice the perpendicular distance between the SLM and the mirror (12.5mm), plus some additional length due to the 5.011^o^ angle of incidence. A Microsoft Excel spreadsheet (WavelengthChange.xlsx) for calculating the parameters is given in the Supplemental Material as an example.

| **λ (nm)** | **SMF array to first plane distance (mm)** | **Plane-to-plane distance (mm)** |
| --- | --- | --- |
| 2100 | 14.905 | 18.702 |
| 2000 | 15.650 | 19.638 |
| 1620 | 19.321 | 24.244 |
| ***1565*** | ***20*** | ***25.096*** |
| 1550 | 20.194 | 25.339 |
| 1480 | 21.149 | 26.537 |
| 1310 | 23.893 | 29.981 |
| 1260 | 24.841 | 31.171 |
| 1064 | 29.417 | 36.913 |
| 980 | 31.939 | 40.077 |
| 904 | 34.624 | 43.446 |
| 850 | 36.824 | 46.206 |
| 810 | 38.642 | 48.488 |
| 800 | 39.125 | 49.094 |
| 780 | 40.128 | 50.353 |
| 660 | 47.424 | 59.508 |
| 632 | 49.525 | 62.144 |
| 532 | 58.835 | 73.825 |
| 473 | 66.173 | 83.034 |
| 405 | 77.284 | 96.976 |

**Supplementary Table 2 |**  Plane spacing for pre-calculated masks as a function of wavelength

*Using pre-calculated masks with different basis dimensions*

The pre-calculated masks can also be used with input/output bases that have been resized, by appropriate scaling of the distance between planes. In this case, the distance between planes scales with the square of the scaling of the input/output bases’ dimensions. For example, if the original basis dimensions of MFD=60μm (input SMFs) and MFD=400μm (output HGs) are doubled to 120μm and 800μm respectively, the mask dimensions must be doubled in size and the spacing between planes must increase by a factor of 4. From 25.096mm to 100.384mm.

**Supplementary References**

1. Carpenter, J., Eggleton, B. J. & Schröder, J. Observation of Eisenbud–Wigner–Smith states as principal modes in multimode fibre. *Nat Phot.* **9,** 751–757 (2015).

2. Fontaine, N. K. *et al.* Characterization of Space-Division Multiplexing Systems using a Swept-Wavelength Interferometer. in *Optical Fiber Communication Conference/National Fiber Optic Engineers Conference 2013* OW1K.2 (Optical Society of America, 2013). doi:10.1364/OFC.2013.OW1K.2

3. Carpenter, J., Eggleton, B. J. B. J. & Schröder, J. Complete spatiotemporal characterization and optical transfer matrix inversion of a 420 mode fiber. *Opt. Lett.* **41,** 5580–5583 (2016).

4. Carpenter, J., Eggleton, B. J. B. J. J. & Schröder, J. 110X110 Optical Mode Transfer Matrix Inversion. *Opt. Express* **22,** 96 (2014).

5. Goodman, J. W. & Lawrence, R. W. Digital image formation from electronically detected holograms. *Appl. Phys. Lett.* **11,** 77–79 (1967).

6. Saleh, B. E. A. & Teich, M. C. *Fundamentals of Photonics*. (John Wiley & Sons, Inc., 1991). doi:10.1002/0471213748

7. Carpenter, J., Thomsen, B. C. & Wilkinson, T. D. Degenerate mode-group division multiplexing. *J. Light. Technol.* **30,** 3946–3952 (2012).

8. Forbes, A., Dudley, A. & McLaren, M. Creation and detection of optical modes with spatial light modulators. *Adv. Opt. Photonics* **8,** 200–227 (2016).

9. Flamm, D., Naidoo, D., Schulze, C., Forbes, A. & Duparré, M. Mode analysis with a spatial light modulator as a correlation filter. *Opt. Lett.* **37,** 2478–2480 (2012).

10. Yao, A. M. & Padgett, M. J. Orbital angular momentum: origins, behavior and applications. *Adv. Opt. Photonics* **3,** 161–204 (2011).

11. Allen, L., Beijersbergen, M. W., Spreeuw, R. J. C. & Woerdman, J. P. Orbital angular momentum of light and the transformation of Laguerre–Gaussian laser modes. *Phys. Rev. A* **45,** 8185–8189 (1992).

12. Leon-Saval, S. G., Argyros, A. & Bland-Hawthorn, J. Photonic lanterns. *Nanophotonics* **2,** 429 (2013).

13. Leon-Saval, S. G. *et al.* Mode-selective photonic lanterns for space-division multiplexing. *Opt. Express* **22,** 1036–1044 (2014).

14. Morizur, J.-F. *et al.* Programmable unitary spatial mode manipulation. *J. Opt. Soc. Am. A* **27,** 2524–2531 (2010).

15. Abramochkin, E. & Volostnikov, V. Beam transformations and nontransformed beams. *Opt. Commun.* **83,** 123–135 (1991).

16. Beijersbergen, M. W., Allen, L., van der Veen, H. E. L. O. & Woerdman, J. P. Astigmatic laser mode converters and transfer of orbital angular momentum. *Opt. Commun.* **96,** 123–132 (1993).

17. Volostnikov, E. G. A. and V. G. Generalized Gaussian beams. *J. Opt. A Pure Appl. Opt.* **6,** S157 (2004).

18. Berkhout, G. C. G., Lavery, M. P. J., Courtial, J., Beijersbergen, M. W. & Padgett, M. J. Efficient Sorting of Orbital Angular Momentum States of Light. *Phys. Rev. Lett.* **105,** 153601 (2010).

19. Mirhosseini, M., Malik, M., Shi, Z. & Boyd, R. W. Efficient separation of the orbital angular momentum eigenstates of light. *Nat. Commun.* **4,** 2781 (2013).

20. Dudley, A. *et al.* Efficient sorting of Bessel beams. *Opt. Express* **21,** 165–171 (2013).

21. Fontaine, N. K. *et al.* Laguerre-Gaussian mode sorter supplementary information. (2019). doi:10.14264/uql.2019.81
